# Light-driven plasmonic microrobot for nanoparticle manipulation

**DOI:** 10.1038/s41467-025-57871-x

**Published:** 2025-03-15

**Authors:** Jin Qin, Xiaofei Wu, Anke Krueger, Bert Hecht

**Affiliations:** 1 Nano-Optics and Biophotonics Group, Experimentelle Physik 5, Physikalisches Institut, Universität Würzburg, Am Hubland, Würzburg, Germany; 2 Leibniz Institute of Photonic Technology, Albert-Einstein-Straße 9, Jena, Germany; 3 Institute of Organic Chemistry, University of Stuttgart, Pfaffenwaldring 55, Stuttgart, Germany

**Keywords:** Optical manipulation and tweezers, Nanophotonics and plasmonics

## Abstract

Recently light-driven microdrones have been demonstrated, making use of plasmonic nanomotors based on directional resonant chiral light scattering. These nanomotors can be addressed individually, without requiring the tracking of a focused laser, leading to exceptional 2D maneuverability which renders microdrones a versatile robotic platform in aqueous environments. Here, we incorporate a light-operated manipulator, a plasmonic nano-tweezer, into the microdrone platform, rendering it a microrobot by enabling precise, all-optical transport and delivery of single nanoparticles suspended in solution. The plasmonic nano-tweezer consists of a resonant cross-antenna nanostructure exhibiting a central near-field hot spot, extending the ability of traditional optical tweezers based on focused laser beams to the trapping of nanoparticles. However, most of plasmonic nano-tweezers are fixed to the substrates and lack mobility. Our plasmonic microrobot utilizes circularly polarized light to control both motors and for stable trapping of a 70-nanometer fluorescent nanodiamond in the cross-antenna center. Complex sequences of microrobot operations, including trap-transport-release-trap-transport actions, demonstrate the microrobot’s versatility and precision in picking up and releasing nanoparticles. Our microrobot design opens potential avenues in advancing nanotechnology and life sciences, with applications in targeted drug delivery, single-cell manipulation, and by providing an advanced quantum sensing platform, facilitating interdisciplinary research at the nanoscale.

## Abstract (web summary)

A 3.5 μm robotic device featuring plasmonic nanomotors and tweezer can deliver, capture, and release a single 70 nm nanodiamond. In this study, the authors develop and demonstrate such a microrobotic device.

## Introduction

Light-driven microdrones have recently emerged as a novel micro-robotic platform, providing unprecedented control over movement and manipulation within microscopic environments^1^. As opposed to the use of standard optical tweezers, microdrones offer direct control over all three degrees of freedom of 2D motion, using individually addressable plasmonic nanomotors, positioning them as an advanced micro-robotic platform for a broad spectrum of applications.

In nanotechnology, a critical challenge is the nanometer-precise transport and delivery of cargo, particularly nano-sized particles, in liquid media. Traditional optical tweezers^2–4^, have demonstrated proficiency in handling micro-objects but their capabilities are significantly limited when dealing with nanoparticles due to limitations of the trapping potential imposed by the diffraction limit and a lack of precise control over the nanoparticle’s orientation. Techniques to induce particle rotation, such as utilizing rotating linear polarization^5^ or chiral absorption of circularly polarized light^6,7^, have been explored, but these methods often compromise nanoscale precision. Other methods necessitate unwieldy configurations, e.g., the attachment of large handles to be controlled in a multi-trap configuration.

Plasmonic tweezers, offering strong, localized trapping potentials, have been recognized as a viable alternative for the trapping of small particles^8–11^. However, a critical issue is the restricted mobility of most plasmonic tweezers, which are fixed to a substrate, thus limiting their potential for cargo transport^12–20^ and precise orientation control of nanoparticles. Quidant et al. have tackled this problem by creating a plasmonic tweezer at the apex of a tapered metal-coated glass fiber^21^. Yet, this method still provides limited local maneuverability compared to a light-driven microdrone. Plasmonic holograms^22^ have been used to introduce subwavelength positioning precision, but the spatial range is very limited. Ghosh et al. have introduced the concept of a plasmonic tweezer trapped in a conventional tweezer. Although providing some additional mobility, the used disc-shaped plasmonic tweezer lacks trapping precision and the system offers only rudimentary control over the particle orientation^23^.

Beyond optical forces, alternative mechanisms, such as magnetic^24^, thermal-hydrodynamic^25–28^,and electro-thermo-plasmonic forces^29,30^ have been investigated to extend the long-range transport capabilities of optical tweezers. Yet, they require special working environments or particle materials. Thus, it remains challenging to deliver a single suspended nanoparticle to a particular position with correct orientation while being firmly trapped during transport.

**Fig. 1:**
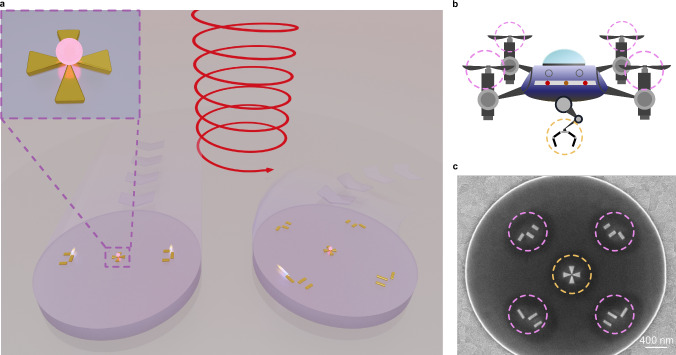
Schematic illustration of the microrobot. **a** Two kinds of microrobots, exhibiting either two or four motors and a tweezer structure, are optically driven in aqueous environment using an unfocused circularly polarized laser beam (980 nm). By adjusting the intensity (${S}_{0}$) and helicity (${S}_{3}$) of the laser beam, the microrobot can be made to follow a specific path (indicated by the faint arrows) and capture a single suspended nanodiamond particle observable via its fluorescence. Inset top left: Magnified artistic view of a nanodiamond particle being trapped by the gold cross-antenna. **b** A cartoon image depicts the main idea of the microrobot concept, which combines individually addressable motors providing precise steering and an independent manipulator. **c** SEM image of the fabricated microrobot. The dashed circles in **b** and **c** indicate the corresponding structures marked by the purple and yellow dashed circles in **b**. Scale bar: 400 nm.

In this paper we integrate the unique capabilities of light-driven microdrones with the possibility of accurate nanoscale cargo transport and delivery, particularly focusing on the manipulation of single suspended nanoparticles. We supplement our microdrone design with a resonant gold cross-antenna which acts as a plasmonic tweezer element (Fig. 1)^31^. This cross-antenna harnesses the power of the circularly polarized incident laser beams to create a strong localized field at its center, facilitating the precise trapping of individual fluorescent nanodiamonds with an average diameter of 70 nm via gradient forces. We demonstrate intricate interactive manipulation sequences, including trapping, transport, release, and re-trapping of nanodiamonds, underscoring the transformative potential of our approach for applications ranging from nanoscale cargo transport to drug delivery and localized quantum sensing.

## Results

The microrobot consists of two main parts: the microdrone and the tweezer structure, integrated in a single platform, shown in Fig. 1. The microdrone design is similar to what we have reported previously^1^. It has a transparent, rigid disk-shaped body made of hydrogen silsesquioxane (HSQ), with a diameter of approximately 3.5 μm, a height of about 150 nm and a mass of 3.8 pg. Several plasmonic antennas (indicated by the purple dashed circles in Fig. 1c) are included to serve as plasmonic nanomotors. They are designed to exhibit resonant chiral response to circularly polarized photons. Attached at the center of the microdrone’s body is a gold cross-antenna^31^, which serves as plasmonic tweezer. The plasmonic tweezer is created in a single fabrication step together with the microdrones’ plasmonic nanomotors (Supplementary Information S3). We have ensured that there is enough space between the motors and the tweezer structure, approximately 1 μm, to prevent interference. Each of the plasmonic nanomotors can be controlled individually using an unfocused circularly polarized laser beam. The laser’s intensity (represented by the Stokes parameter ${S}_{0}$) controls the speed of movement by increasing or decreasing the number of directionally scattered photons per unit time and thus the photon recoil, while changing the light helicity (represented by the Stokes parameter ${S}_{3}$) alters the thrust direction. It is important to note that the tweezer’s trapping capability is sensitive only to the ${S}_{0}$ component and thus the setup allows us to steer the microrobot flexibly by adjusting the ${S}_{3}$ component without affecting the trapping effect.

**Fig. 2:**
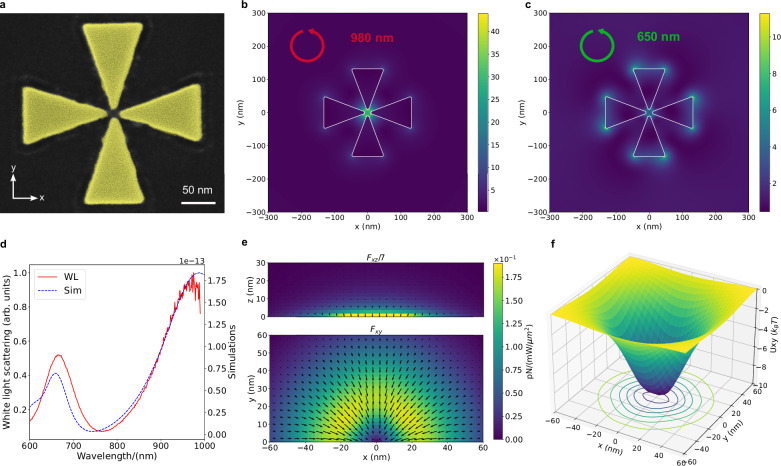
Characterization of the cross-antenna plasmonic tweezer element. **a** SEM image of the gold cross-antenna tweezer element. Scale bar: 50 nm. **b**, **c** Electric near-field enhancement of cross-antenna at 980 nm **b** and 650 nm **c** with counterclockwise polarized light. **d** White light scattering spectrum (red) and FDTD simulation (blue). **e** Out of plane (upper panel) and in-plane (lower panel) optical force distribution map calculated using COMSOL. The ${{{{\bf{F}}}}}_{{xy}}$(${{{{\bf{F}}}}}_{{xz}}$) map is obtained by scanning the nanodiamond position in a simulation in the xy(xz) plane with a resolution of 2 nm within an area of 120 nm by 60(30) nm. Out-of-plane optical forces (${F}_{{xz}}$) are divided by a factor of 7 to fit the shared color bar. The direction of the force is indicated by the black arrows. **f** The trapping potential well is evaluated using the laser intensity employed in the experiments (3 ${mW}/{\mu m}^{2}$). The depth of the trapping potential is about 10${k}_{B}T$.

The gold cross-antenna is constructed from four gold triangle structures, each with a base-side length of approximately 90 nm, a height length of about 120 nm, and a thickness of roughly 50 nm. The tip-to-tip gap size of each opposing triangle pair is approximately 20 nm. The SEM image in Fig. 2a provides a visual representation of this structure. These structures were fabricated using the outline milling technique with a Helium microscope (HIM) from a single-crystalline gold platelet, as detailed in Supplementary Information S3.

The geometry of the gold cross-antenna has been optimized to resonate in water with circularly polarized light at a vacuum wavelength of 980 nm. This resonance is verified by a white light scattering measurement (Fig. 2d), which displays two peaks matched by numerical simulations. The lower-energy peak corresponds to a mode with electric near-field enhancement concentrated within the gap region, as illustrated in Fig. 2b. The higher-energy peak is associated with another mode localized at the base region of the equilateral triangles (Fig. 2c), which does not pertain to the trapping potential.

To determine the time-averaged gradient trapping force exerted on a nanodiamond particle close to the cross-antenna, we integrate the time-averaged Maxwell’s Stress Tensor (MST) ${{\langle }}{{{\bf{T}}}}{{\rangle }}$ over the nanodiamond’s surface. To visualize the trapping forces, Fig. 2e presents vector maps of the in-plane (${{{{\bf{F}}}}}_{{xy}}$) and the out-of-plane (${{{{\bf{F}}}}}_{{xz}}$) optical gradient forces. These maps are generated by shifting the nanodiamond’s position within the central trapping region (120 nm × 60 nm for in-plane, 120 nm × 30 nm for out-of-plane) within the corresponding planes (xy and xz, respectively). The presence of a strong out-of-plane optical gradient force, ${F}_{{xz}}$, is evident, directing the nanodiamond towards the antenna’s center, as illustrated in the upper panel of Fig. 2e. In contrast, the in-plane forces ${{{{\bf{F}}}}}_{{xy}}$ exhibit a complex behavior, affecting the nanodiamond’s trapped position. The maximum optical gradient force is found around the tips of the triangles, consistently directed towards the center (lower panel of Fig. 2e). This trapping behavior can be conceptually understood as the nanodiamond being drawn toward the antenna’s center regardless of its approach direction. To assess the depth of the trapping potential well, we integrate the forces along a radial path, yielding $U\left({{{{\bf{r}}}}}_{0}\right)=-{\int }_{-\infty }^{{{{{\bf{r}}}}}_{0}}{{{\bf{F}}}}\left({{{\bf{r}}}}\right)d{{{\bf{r}}}}$. Figure 2f illustrates the presence of a stable optical trap, with the trapping potential energy ($U$) reaching $10{k}_{B}T$ for a laser intensity of 3 ${mW}/{\mu m}^{2}$.

To assess the trapping capability of the gold cross-antenna, a static plasmonic tweezer was fabricated and a trapping experiment was conducted. To this end, the tweezer structure was fabricated directly onto a coverslip (see Methods) and placed within a pure-water cell. This cell contained water-dispersed nanodiamonds with an average diameter of 70 nm at an appropriately low concentration. The nanodiamond particles were engineered to possess multiple color centers to enhance fluorescence brightness, and their diameter was confirmed through atomic force microscopy (AFM) scanning (Supplementary Information S1).

**Fig. 3:**
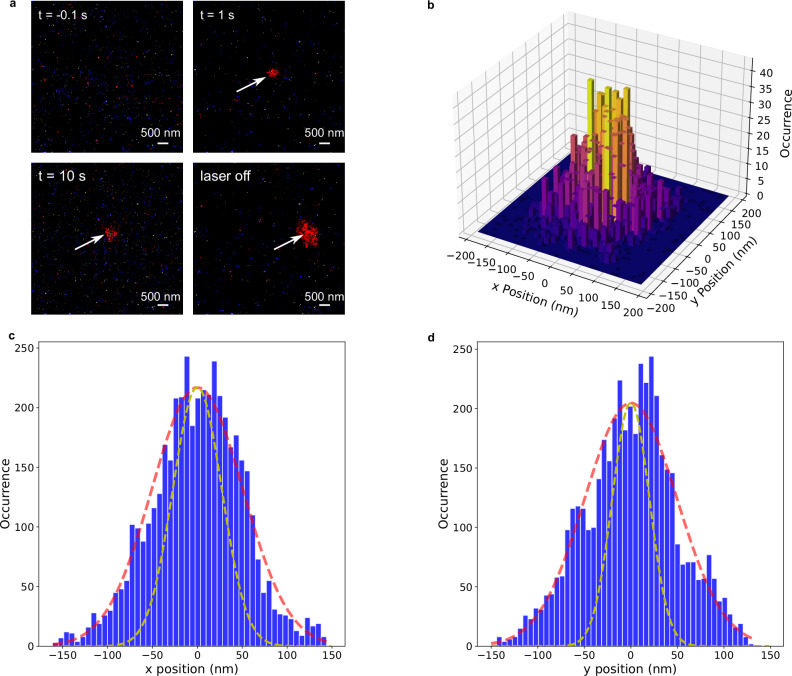
Static tweezer experiments and determination of trapping stiffness. **a** Frame sequences extracted from the video (Supplementary Movie 1). Trapping experiments are performed using an unfocused laser beam. When the laser is off ($t=-0.1s$), no fluorescence is observed. Upon turning on the laser, a single nanodiamond moves close to the tweezer and gets trapped (indicated by the red spot in frames at $t=1s$ and $t=10s$). Subsequently, when the laser is turned off, the nanodiamond quickly disappears, indicating no adhesion between the nanodiamond and the tweezer. Scale bar: 500 nm. **b** Statistical analysis of the fluorescence position extracted from the video. **c**, **d** Histograms depicting the distribution of the trapped nanodiamond’s position in the x and y directions. The red dashed lines represent Gaussian fits. The yellow dashed lines represent the instrument response obtained by detecting and analyzing a single nanodiamond directly adhered to the coverslip under otherwise identical illumination and detection conditions (Supplementary information S9).

A continuous-wave infrared laser diode with a wavelength of 980 nm was employed to create a laser spot with a diameter of 10 μm (FWHM) using a microscope objective (NA: 0.5). Circular polarization was achieved by adding a quarter-wave plate. Additionally, a green laser (532 nm, laser intensity: 0.002 ${mW}/{\mu m}^{2}$) was introduced to excite the diamond color centers, operating at a wavelength far from the resonances of the cross-antenna. Fluorescence was collected by a high-numerical-aperture oil-immersion microscope objective (NA: 1.49) and recorded using a CMOS camera (DFK, The Imaging Source Europe GmbH) at a frame rate of 30 frames per second (Fig. S2b). Figure 3a presents sequential images extracted from the static tweezer experiments showing an area of the sample with the plasmonic tweezer structure at its center. Prior to activating the infrared laser, no fluorescence is discernible in the image. Upon activating the infrared laser, a single nanodiamond particle approaching the structure is effectively trapped by the cross-antenna tweezer, evident from the red spot in Fig. 3a (top-right and bottom-left panels). When the laser is deactivated, the nanodiamond promptly departs (bottom-right panel). To determine the trapping stiffness of the plasmonic tweezer structure, video sequences of the static tweezer experiment with a trapped nanodiamond were processed, and the centroids of the red fluorescent spots were extracted (Supplementary Information S4). Analyzing the distribution of detected nanodiamond positions reveals that the most frequent trapping positions are concentrated within the antenna center region, as depicted in the 3D histogram in Fig. 3b. By employing the expressions ${k}_{x}={k}_{B}T/{{\langle }}{x}^{2}{{\rangle }}$ and ${k}_{y}={k}_{B}T/{{\langle }}{y}^{2}{{\rangle }}$^32^, where ${{\langle }}{x}^{2}{{\rangle }}$ and ${{\langle }}{y}^{2}{{\rangle }}$ represent the mean square displacements in the x and y directions from the equilibrium point and assuming a harmonic trapping potential, the trapping stiffnesses ${k}_{x}$ and ${k}_{y}$ in the x- and y- directions were extracted. We found ${k}_{x}=$1.61 fN/nm and ${k}_{y}=$1.68 fN/nm, respectively, aligning well with values reported in previous studies^7,10,11^. However, it is important to acknowledge that in our case, the trapping stiffness is somewhat underestimated due to the significant influence of the instrument response, i.e. the position uncertainty of the single nanodiamond particle in each movie frame, on the fluorescence distribution, indicated by the yellow dashed line in Fig. 3c, d. A more comprehensive discussion on this matter is provided in Supplementary Information S9.

The integration of the gold cross-antenna into the microrobot body is performed in two distinct microrobot configurations: a two-motor and a four-motor design (Fig. S3). The working concept of the plasmonic nanomotors is based on directed photon scattering upon illumination with clockwise (CW) and counterclockwise (CCW) polarized light, as explained in Supplementary Information S5. This switches the near-field intensity distribution of the nanomotors which leads to distinct changes in the photon emission patterns^1^. The lateral optical thrust force driving the microrobots originates from directional scattering of light causing a photon recoil. In Fig. S4a–d, we illustrate the chiral response of the symmetric nanomotor used in the two-motor microrobot configuration. When changing the light helicity from CW to CCW, the electric near-field distribution and the corresponding far-field radiation pattern will be mirrored at the xy plane, resulting in a switching of the lateral optical force from the -x to the +x direction. However, the asymmetric nanomotor design (Fig. S4e–h) used in the four-motor configuration exhibits good directivity and generates lateral optical forces only for CCW polarized light. For CW polarized light, the resulting symmetric far-field radiation pattern leads to a nearly zero net lateral optical force. These properties suggest that the actuation pattern of the four-motor design is expected to induce rotational motion, as the optical torque remains constant regardless of the microrobot’s rotation angle. Due to the presence of two identical nanomotors, the two-motor design is theoretically expected to exhibit a linear translational motion, as both nanomotors scatter light in the same direction with equal efficiency. Yet, the spin momentum transfer from circularly polarized photons upon absorption provides a non-zero background torque contribution independent of the nanomotor design which can lead to a slight curvature of observed trajectories. It is important to note that in all cases all gold structures are not entirely enclosed within the microrobot’s body to avoid a thick encapsulation layer on top of the plasmonic tweezer structure, which would diminish its trapping capability.

**Fig. 4:**
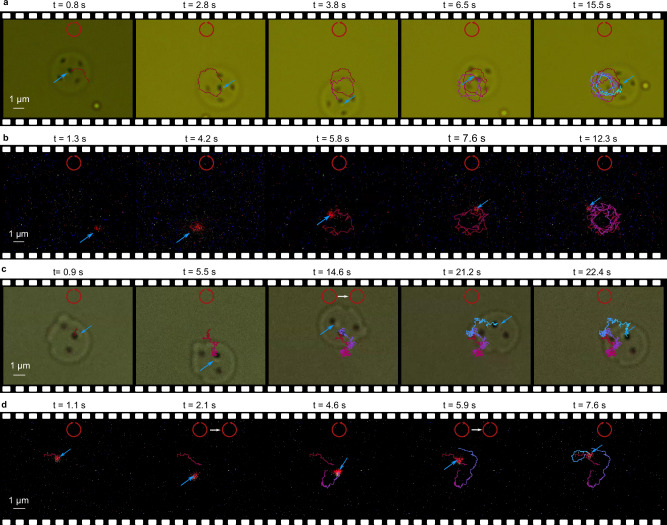
Manipulation of a single suspended nanodiamond using Microrobots. **a** Bright-field (Supplementary Movie 2) and **b** fluorescence (Supplementary Movie 3) frame sequences depict the spiral motion of the microrobot with a trapped nanodiamond. The light properties are indicated in the top of each frame, while the nanodiamond position is denoted by a blue arrow. The trajectories are obtained by analyzing the center position of the microrobot (bright-field) and the fluorescence. It is important to note that the large spherical object observed in **a** is a contamination and not a single nanodiamond, as it lacks fluorescence. **b** The trapping dynamics are displayed in fluorescence. In the first frame, a single nanodiamond approaches the microrobot. After some time, it is captured by the tweezer (second frame), and then it follows a spiral motion along with the microrobot, as depicted in the trajectories. **c**, **d** Translational motions of the microrobot with the trapped nanodiamond (Supplementary Movie 4 and Supplementary Movie 5)) are displayed. In the third frame of **c** and second (fourth) frame of **d**, the laser’s helicity is modified, resulting in a corresponding change in the microrobot’s motion. During the helicity switching, the laser is temporarily blocked by the wave-plate mount, leading to the release of the nanodiamond. Subsequently, the nanodiamond is recaptured again, which indicates a trap-transport-release-trap-transport behavior.

To perform mobile trapping experiments, the microrobots are liberated from the substrate and allowed to float in a water solution, in which the nanodiamonds are sparsely dispersed (Methods). Upon activation of the infrared laser, it exerts light pressure vertical to the circular top surface of the microrobot, causing it to be softly pushed against the substrate. However, the microrobot does not adhere to the substrate due to the presence of surface charges. In Fig. 4, we illustrate the trapping effects and the long-range transport behavior through four exemplary processed video frames in both bright field illumination and fluorescence.

Figure 4a, b depict four-motor microrobots, as shown in Fig. 1c, executing a spiral trajectory while demonstrating the trapping effects. CCW circularly polarized light is applied (intensity: 3 ${mW}/{\mu m}^{2}$, spot size (diameter): 10 μm), resonantly addressing one of the four motors to generate optical torque via photon recoil. Before trapping, the microrobot describes a narrow circle in CCW direction with an angular velocity of 2.96 rad/s, and no red fluorescence is observed. However, when a single nanodiamond approaches the tweezer (as seen in the first frame of Fig. 4b), it becomes subject to the optical gradient force and is ultimately trapped, as indicated by the blue arrow in Fig. 4a, b. Despite the microrobot’s spiral motion performing multiple turns, the nanodiamond remains securely attached to the tweezer structure. Supplementary Movie 6 offers more comprehensive insights into trapping dynamics, including the process of nanodiamond trapping by the tweezer and the steps involved in releasing and re-trapping the nanodiamond.

Furthermore, the microrobot exhibits a high degree of controllable maneuverability, as illustrated in Fig. 4c, d, demonstrating a trap-transport-release-trap-transport sequence. For this purpose, the design is modified to a two-motor configuration, as evidenced in the SEM image in Fig. S3. In principle, altering the light’s helicity should induce forward-backward translation. However, curved trajectories are observed instead of the expected straight paths, which can be attributed to superimposed rotational motion due to two effects. Firstly, this motion can result from the intrinsic spin momentum transfer caused by absorption of circularly polarized light, leading to a non-zero optical torque that inevitably induces rotation. Secondly, a gradient in the  light intensity between the two motors can also contribute to the generation of a torque. In the initial two frames of Fig. 4c, the microrobot, carrying a single nanodiamond, achieves a translation velocity of 4.55 μm/s (Supplementary Information S7). In the middle frame, the helicity of the circularly polarized light is reversed from CCW to CW by inserting a half-wave plate. During this transition, the laser is momentarily blocked by the mount of the waveplate, causing the nanodiamond to be released from the trap while remaining in proximity. Subsequently, the microrobot alters its direction, recapturing the nanodiamond. It then carries the nanodiamond to another position, and even back to the origin. In Fig. 4d, the helicity of the circularly polarized light undergoes two alterations in the second and fourth frames, demonstrating multiple trap-transport-release-trap-transport sequences.

Generally, two lasers with different wavelengths can be used to control the motion of the microrobot, as demonstrated in the microdrone system, enabling us to achieve all three degrees of freedom of 2D motion. It is also possible to steer the motion of the microrobot with two laser beams while maintaining the trapping effect. In this scenario, an additional CW polarized laser with a wavelength of 830 nm was used, which resonantly addressed another nanomotor without affecting the trapping effects, generating optical torque that countered the torque produced by the 980 nm laser (CCW polarized laser). When these two optical torques were well balanced, the remaining optical force propelled the microrobot into a linear motion.

**Fig. 5:**
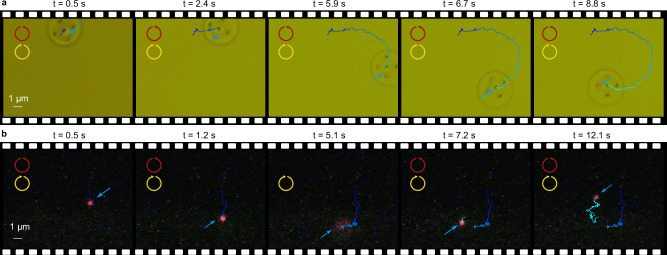
Microrobot manipulation with two laser beams. **a** Bright-field (Episode 1 in Supplementary Movie 7) and **b** fluorescence (Episode 2 in Supplementary Movie 7) frame sequences showing translational motion of the 4-motor microrobot driven by two laser beams. The nanodiamond and red fluorescence spots are indicated by blue arrows. CCW polarized laser (980 nm, red circular arrow, intensity: 1.3 ${mW}/{\mu m}^{2}$) and CW polarized laser (830 nm, yellow circular arrow, intensity: 1.4 ${mW}/{\mu m}^{2}$) were applied simultaneously. Taking the second frame in **a** as an example, the optical force is directed towards the side where two different motor designs are present (right side). When the microrobot reaches the margin of the laser spot, the generated optical force vanishes. Driven by Brownian motion, the microrobot changes its orientation and continues the translation motion, resulting in the microrobot hovering inside the laser spot while maintaining the translational motion and capturing the nanodiamond. **b** The nanodiamond can also be released by temporarily reducing the intensity of the 980 nm laser to zero (third frame). Once the laser intensity is restored, the nanodiamond is recaptured. Scale bar: 1 $\mu m$.

Additionally, the microrobot demonstrated the ability to capture a nanodiamond and transport it to a different position, as shown in the bright-field and fluorescence frame sequences in Fig. 5. Due to the finite size of the laser spots, the microrobot moved along a linear trajectory until it reached the edge of the laser spot. At that point, the decreased laser intensity caused the microrobot to turn and continue moving, as illustrated in Fig. 5a. Moreover, releasing the nanodiamond was achieved, as depicted in the third frame of Fig. 5b, by temporarily blocking the 980 nm laser. The nanodiamond was then recaptured when the 980 nm laser intensity was restored. This demonstration highlights the potential for separating steering and trapping effects in microrobots by employing different laser wavelengths.

Ideally, to fully decouple the optical thrust force from the plasmonic trapping effect, lasers with wavelengths of 830 nm and 980 nm could be used exclusively to control nanomotors, while a third laser, such as one with a wavelength of 1064 nm, could independently control the plasmonic tweezer effect without influencing the microrobot’s motion. However, there are challenges associated with using multiple laser beams. First, achieving uniform laser intensity in regions where multiple beams overlap can be difficult. As a result, high local intensity in overlapping regions could locally increase the optical force or torque acting on the microrobot, causing it to suddenly accelerate and lose its grip on the nanodiamond. Second, plasmonic thermal effects may become more pronounced, especially when three laser beams are employed. The heating effects can be mitigated by replacing the microrobot’s body material with alternatives that have higher thermal conductivity, such as silicon carbide or diamond. Additionally, microstructures like fins or pillars can be fabricated on the microrobot’s body to increase the surface area and enhance heat dissipation.

## Discussion

Our microrobot offers a range of intricate manipulation scenarios, including: (i) Positioning and trapping. The microrobot can be navigated to a specific location and effectively trap a nanoparticle. (ii) Releasing cargo at a specific position. By momentarily obstructing the laser, the nanoparticle can be released precisely at a designated location. (iii) Recapture and transport of cargo. The microrobot can recapture a released nanoparticle and transport it to another chosen spot. The orientation of the particle may also be controlled via the orientation of the microrobot.

In practical applications, the recapturing step is not always necessary. Instead, blocking the laser for an extended period of time enables the nanodiamond to diffuse further away. On the other hand, employing an electro-optic (EO) modulator to switch the helicity of circularly polarized laser can adjust the motion of the microrobot without losing the trapping effect. In Fig. S5a, b, we demonstrate that by utilizing several EO modulators, the movement direction of the microrobot can be rapidly adjusted without losing the plasmonic trapping effects. For instance, the microrobot can be driven to move back and forth or even retrace its path along the same route (Episode 1–2 in Supplementary Movie 8). In addition, with higher laser power, we can extend the working dynamic range of microrobot as shown in Fig. S5c. In this demonstration, our microrobot also captures two nanodiamonds simultaneously (Episode 3 in Supplementary Movie 8). Regarding the potential to scale up the trapping capabilities of microrobots, we expect that the fabrication of several tweezer structures should make it possible to load multiple particles, provided the microrobot’s body is large enough.

Additional laser beams can be introduced to separate the motion steering and trapping effects. As demonstrated in Fig. 5, two laser beams with different wavelengths are used to steer the microrobot in a linear motion while capturing a single nanodiamond. In this case, the motion of the microrobot is controlled by two laser beams, while the plasmonic tweezer is only resonantly addressed by one of them. This approach offers opportunities by introducing additional laser beams for independent control of the tweezer effect. Besides, with more advanced motor designs capable of generating out-of-plane optical forces, microrobot motion can be extended from 2D to 3D while maintaining trapping effects. Furthermore, in Fig. S7, we present the position accuracy of nanodiamonds captured by microrobots, which is predominantly influenced by Brownian motion acting on the microrobots. This occurs because, at present, the microrobot itself is not subject to any trapping potential to counteract Brownian motion. However, with the implementation of an active feedback system to control the position thereby compensating for Brownian motion, more precise nanoscale particle delivery will become feasible.

The microrobot’s versatile transport and trapping capabilities hold significant promise for a variety of exciting applications. For instance, in the realm of biology, it can capture and transport nano-sized entities such as drug-containing vesicles and proteins to specific targets. Such drug delivery experiments can be implemented both in vitro and potentially in vivo provided that the drag force is sufficiently small. For in vivo applications, studies have shown that applying special absorption molecules to the skin, such as FDA-approved tartrazine, can temporarily render it transparent^33^. Besides, transparent animals can be used for such applications. To give a first glimpse into biological applications, in Fig. S5d, we demonstrate to use our microrobots to capture, transport, and release a rod-shaped bacterium (*Pseudomonas*, Episode 4 in Supplementary Movie 8). Additionally, in the field of local quantum sensing, the microrobot could be used to capture a nanodiamond containing a single color center, which then may serve as a freely movable single-photon source or a magnetometry sensor^34^. In detail, to facilitate efficient coupling of microwave radiation into the color center, a dedicated on-chip microwave antenna should be fabricated on the bottom coverslip. The separation distance between the bottom coverslip and the microrobot is on the order of several hundred nanometers, ensuring optimal coupling efficiency. Subsequently, a standard optically detected magnetic resonance (ODMR) setup could be employed to probe the color center’s spin state dynamics. By manipulating the microrobot’s motion, this platform can be used to sense local magnetic field distribution at any desired position in liquid environment.

It is important to note that thermal effects play a substantial role in the trapping characteristics of the microrobot due to the absorption of incident light by the plasmonic structure. In our case, heat-induced thermophoresis is recognized as a challenge to trapping since it pushes the nanoparticle away from the hot region to the cold region (Supplementary Information S8). Therefore, an excessively high laser intensity can adversely affect the trapping efficiency. Additionally, it is essential to balance the microrobot’s velocity with its trapping effectiveness. At higher velocities, the drag force acting on the nanodiamond increases, which can lead to the detachment of the trapped nanodiamond.

In summary, our study demonstrates a method for the precise manipulation of a single nanodiamond particle utilizing a microrobot that combines plasmonic motors with a tailored plasmonic nano-tweezer. Employing circularly polarized laser beams without focusing, we achieve control of the microrobot’s trajectory on the nanometer scale and generate a stable trapping potential for 70 nm diameter nanodiamonds. The microrobot exhibits diverse motion capabilities, including spiral and linear translations, while securely holding the particle. We further showcase complex operational sequences involving trapping, transporting, and releasing the nanodiamond, underlining the technology’s applicability in fields such as lab-on-a-chip systems and in vitro biological experiments.

## Methods

### Numerical simulations

The finite-difference time-domain algorithm (Lumerical FDTD Solutions) is used for optimizing the gold cross plasmonic tweezer geometry. The dielectric function of single-crystalline gold is obtained from Olmon et al.^35^. The gold structure is partially embedded into an HSQ body, whose refractive index is 1.4 independent of the wavelength in the range considered. The background media is replaced by water with refractive index of 1.33. Circularly polarized light is applied to excite the system. The wavelength-dependent scattering cross section is extracted to determine the resonance of the antenna. Optical forces are calculated using COMSOL Multiphysics. The refractive index of nanodiamond is set to 2.0. Perfectly matched layers are added to suppress the reflection from the boundary of the simulation volume. Optical forces ${{{\bf{F}}}}$ are extracted by integrating the time averaged Maxwell’s Stress Tensor ${{\langle }}{{{\bf{T}}}}{{\rangle }}$ over the nanodiamond surface by ${{{\bf{F}}}}={\oint }_{S}{{\langle }}{{{\bf{T}}}}{{\rangle }}\cdot \widehat{{{{\bf{n}}}}}{{{\rm{d}}}}s$, where ${{{{\bf{T}}}}}_{{ij}}=\epsilon {\epsilon }_{0}{{{{\bf{E}}}}}_{i}{{{{\bf{E}}}}}_{j}^{*}+\mu {\mu }_{0}{{{{\bf{H}}}}}_{i}{{{{\bf{H}}}}}_{j}^{*}-\frac{1}{2}{\delta }_{{ij}}(\epsilon {\epsilon }_{0}{\left|{{{\bf{E}}}}\right|}^{2}+\mu {\mu }_{0}{\left|{{{\bf{H}}}}\right|}^{2})$^36^. The heat distribution around the gold cross structure is calculated using COMSOL Multiphysics. The parameters used in the simulation can be found in the Table S1.

### Fabrication

Gold structures are fabricated from single-crystalline gold platelets, which are chemically synthesized on glass coverslips^37,38^. The thickness of the platelets is checked by fitting the transmission spectrum obtained by white light illumination of the platelets. After such pre-characterization, a suitable platelet is transferred to a different substrate according to the needs of the experiments. The coverslip used in experiments is thoroughly cleaned by acetone and isopropanol using an ultrasonic bath. To prevent sticking of microdrones to the substrate, the coverslip is bathed at 70 °C in a (2%) Hellmanex III solution for 1 h. For static tweezer experiments, a gold platelet with suitable thickness is transferred to a coverslip. The gold cross structure is fabricated by focused helium ion beam milling (Zeiss Orion NanoFab). Outline milling and a peeling technique are applied to accurately define the gold structure^1^. After that, the structures are placed in a water cell, which consists of two coverslips sealed with a thin PDMS spacer (thickness: 165 μm) with a hole (diameter: 6 mm) at the center. Before sealing, 3.5 μL of water diluted with a very low concentration of nanodiamond is added. For the dynamic trapping experiment, the substrate is changed to a glass substrate with 700 nm ITO coating. A thin layer (100 nm) of HSQ resist is spin-coated on top to yield a planarized ITO surface. After transferring a gold platelet on top of the planarized substrate, the plasmonic motors and plasmonic tweezer structure are fabricated using the outline milling technique. During this step, the pattern geometry is nicely defined by focused helium ion beam milling and the excess surrounding gold layer is removed by peeling off the remaining gold flake (Supplementary Information S3, Fig. S3a, b, d, e). As a next step, the body of the microrobot is defined by applying electron beam lithography (EBL) and wet chemical etching (Supplementary Information S3, Fig. S3c, f). After that, the microrobot is released from the substrate by using hydrochloric acid (HCl) to etch away the underlying ITO layer and the substrate holding microrobots is turned over and used as the top seal of liquid cell. The microrobots are then detached from the substrate using 5 s mild ultrasonification.

### Optical setup and measurements

The white light scattering spectra are recorded by a home-built setup similar to those illustrated in refs. ^39,40^. The only modification is a change of the excitation white-light beam path by adding a polarizer to create polarized light (Supplementary information S2). The static/dynamic tweezer experiments share the same setup as depicted in Fig. S2b. The laser for driving the microrobots are continuous-wave laser diodes (Thorlabs, BL976-PAG900, LD830-SE650). The output power can be adjusted by controlling the diode current via a Labview program. Circularly polarized light is obtained by the combination of linear polarizer and a quarter wave-plate. A removable half wave-plate can be inserted to change the helicity of light. The beam is loosely focused to the sample by a polarization-conserving objective (Zeiss, EC Epiplan-Neofluar 20 x/0.5 Pol). At the same time, a green laser (*λ* = 532 nm) is collimated at the sample with a larger laser spot. The sample is fixed on a piezo-stage, which is capable of generating motions with nanoscale resolution in 5 axes. Images of the sample are collected by a high NA oil immersion objective (NA: 1.49 Nikon, MRD01991 CFI Apochromat 100 x Oil). A CMOS camera (DFK 37AUX252) is used to record the image plane with 30 fps. The excitation laser and the green laser are filtered by respective filters.

### Reporting summary

Further information on research design is available in the Nature Portfolio Reporting Summary linked to this article.

## Supplementary information

Supplementary Information

Description of Additional Supplementary Files

Supplementary Movie 1

Supplementary Movie 2

Supplementary Movie 3

Supplementary Movie 4

Supplementary Movie 5

Supplementary Movie 6

Supplementary Movie 7

Supplementary Movie 8

Reporting Summary

## Data Availability

All data needed to evaluate the conclusions in the paper are present in the paper and/or the Supplementary Materials. The raw data is available at: https://zenodo.org/records/14873413
