# Supplementary material for: Light-driven plasmonic microrobot for nanoparticle manipulation
Source: Nat Commun. 2025 Mar 15;16:2570. doi: 10.1038/s41467-025-57871-x (PMC11910605; doi:10.1038/s41467-025-57871-x)
Supplement: Supplementary file 1 — Supplementary Information [file 41467_2025_57871_MOESM1_ESM.pdf]

Supplementary Information for

Light-driven plasmonic microrobot for nanoparticle manipulation

Jin Qin,[1*] Xiaofei Wu,[2] Anke Krueger,[3] Bert Hecht[1*]

*Corresponding author. jin.qin@uni-wuerzburg.de; hecht@physik.uni-wuerzburg.de

This PDF file includes:

Fig. S1 to S10

Table S1

Notes S1 to S9

Other Supplementary Material for this manuscript includes the following:

Supplementary Movie 1: Static tweezer experiment.

Supplementary Movie 2: Rotation motion of the microrobot with a trapped nanodiamond (Bright field)

Supplementary Movie 3: Rotation motion of the microrobot with a trapped nanodiamond (Fluorescence)

Supplementary Movie 4: Translation motion of the microrobot with a trapped nanodiamond (Bright field)

Supplementary Movie 5: Translation motion of the microrobot with a trapped nanodiamond (Fluorescence)

Supplementary Movie 6: Entire trapping dynamics with microrobot doing rotation motion

Supplementary Movie 7: Manipulation of the microrobot within two wavelengths while maintaining the trapping effects. **Episode 1**: Bright-filed and **Episode 2**: Fluorescence

Supplementary Movie 8: Another 4 demonstrations for showing the capabilities of microrobots. **Episode 1**: A 4-motor microrobot driven by two laser beams with EO modulators (Bright Field). **Episode 2**: Complex maneuverability of a 2-motor microrobot driven with EO modulators (Fluorescence). **Episode 3**: A 2-motor microrobot operating within an extended dynamic range (Fluorescence). **Episode 4**: A microrobot capturing, transporting, and releasing a single bacterium (Bright Field).

Nanodiamond fabrication (S1)

The commercially available fluorescent HTHTP diamond particles (ND-NV-70) from Adamas Nano Corp. were characterized using dynamic light scattering (Malvern zetasitzer Nano) and the particles redispersed using sonication in doubly distilled water. The surface of the diamond particles was found to be mainly oxygen i.e., carboxyl, terminated as shown by infrared spectroscopy. The details can be found in previous studies[1,2]. According to our previous statistics, at least 90 % of the nanodiamonds have a size less than 70 nm[3]. To verify the size of the nano-diamonds, we spin-coated a diluted nanodiamond water solution onto a clean coverslip and used an atomic force microscope (AFM) to measure them, as illustrated in Fig. S1. The apparent lateral size is slightly larger as the actual size, as nanodiamonds can experience some shifts during scanning.

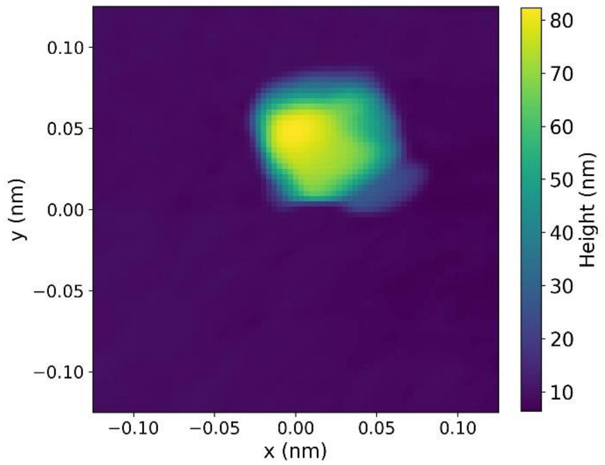

Figure S1: AFM scan of a single Nanodiamond particle on coverslip.

Setup and measurement (S2)

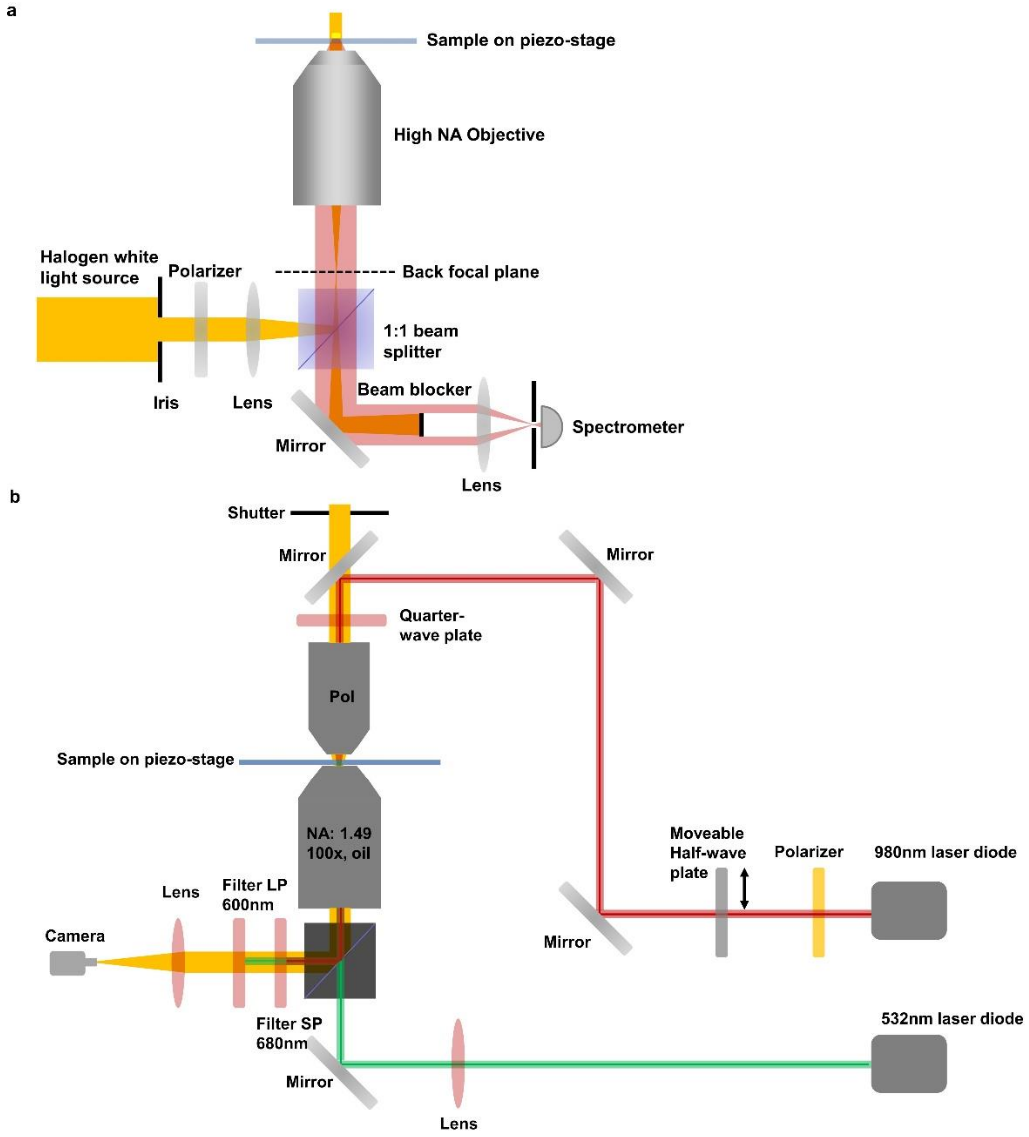

Figure S2: Measurement setup. a. White light scattering measurement setup. **b**. The optical setup for static trapping and dynamic trapping measurements.

In Fig. S2a, the incident white light source is linearly polarized by a polarizer. The excitation beam is blocked completely by a circular beam blocker. Only light scattered by the antenna is collected by the lens and sent to the spectrometer.

In Fig. S2b, the helicity of the incident laser is controlled by a removable half-wave plate. Images are recorded via a high NA objective. The green excitation laser and infrared steering laser are blocked using two optical filters. The white light source is used for illumination and controlled by a shutter. In case of fluorescence, the shutter is off and only fluorescent light is collected and recorded.

Fabrication (S3)

The fabrication of all structures involves focused helium beam milling, where only the outline of the antenna geometry, composed of a point cloud with optimized doses, is cut. This process ensures precise control over critical gap sizes and corner radii, and facilitates an easy one-step flake peel-off process[4]. Fig. S3a-b and d-e demonstrate the well-controlled gap size and corner radius achieved through this fabrication method. Following this, a layer of HSQ with a thickness of approximately 200 nm is spin-coated on top of the structures, and the microrobot body is defined subsequently using an electron beam lithography (EBL) process. Finally, 70 µL 0.5 % of HF solution is applied to the sample surface for 100 s to etch away the bottom HSQ layer used for planarization. In some cases, a second EBL process is employed to further refine the microrobot's geometry. It is crucial that in the end the gold cross antenna is only partially embedded (not completely) into the HSQ body, as depicted in Fig. S3c and f. Complete embedding would result in an exponential decay of the trapping forces with increasing thickness of the encapsulation layer.

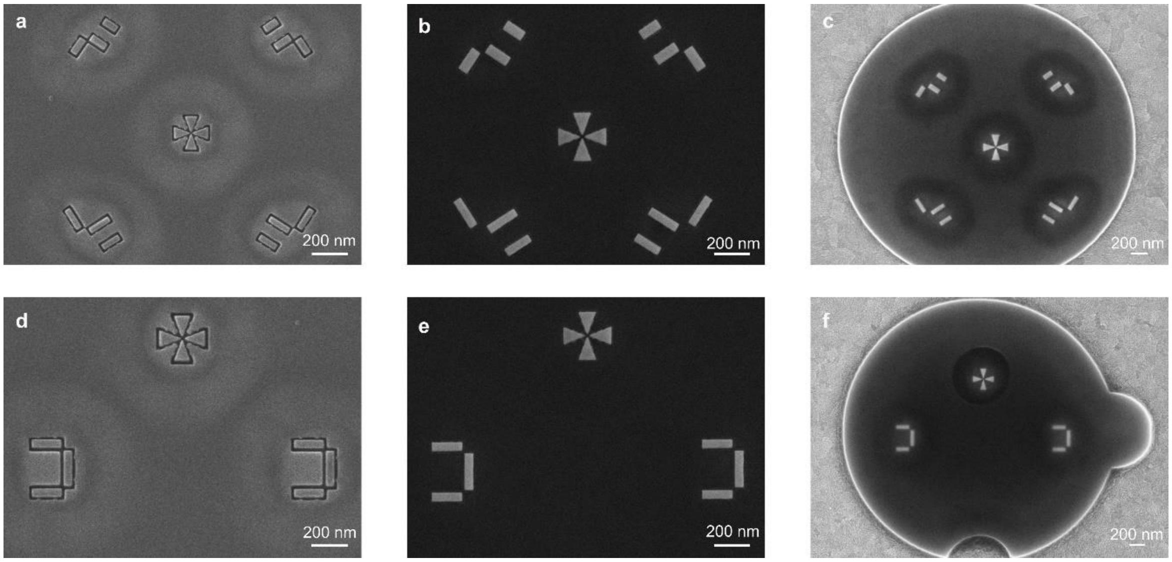

Figure S3: Fabrication process of four-motor (a-c) and two-motor (d-f) microrobot. a and **d**: Scanning helium ion microscope pictures after outline milling. **b** and **e**: Scanning electron microscope pictures after peeling off the gold flake. **c** and **f**: Scanning electron microscope pictures after EBL process. Scale bar: 200 nm.

Image processing (S4)

Video processing is performed by using morphological operation in the OpenCV library (Python). Generally, the colorful image is first binarized by setting a threshold based on the intensity of the target of interest (for example, the fluorescent red dot or motor spot). Then, the erode operation is used to get rid of some small noisy spots. Afterwards, the connected domains are checked and the "mass" center points are extracted as the position

of the nanodiamond in a movie frame. All the fluorescence images and videos are processed with contrast enhanced algorithm.

Actuation pattern (S5)

To induce rotational motion, the optically driven motor is the same as in the four-motor design (Type II) we proposed in the previous publication[4]. One nanomotor is resonantly addressed to generate the thrust force when using one laser (980 nm) with circular polarization. The working principle of plasmonic nanomotors is illustrated as Fig. S4 (e-h). For counterclockwise (CCW) polarized laser, the nanomotor exhibits directional scattering of light, thereby generating optical recoil. In contrast, for clockwise (CW) polarized laser, the nanomotor does not experience optical force due to symmetric light scattering. For translation motion, we employ two motors of the same type (Type I), designed to operate at 980 nm. In this scenario, the direction of photon scattering can be switched by changing the helicity of laser as shown in Fig. S4 (a-d).

Occasionally, curved trajectories are observed instead of the expected straight paths, which can be attributed to superimposed rotational motion due to two effects. Firstly, this motion can result from the intrinsic spin momentum transfer caused by circularly polarized light, leading to a non-zero optical torque that inevitably induces rotation. Secondly, the uneven local light intensity between the two motors can also contribute to the generation of a torque.

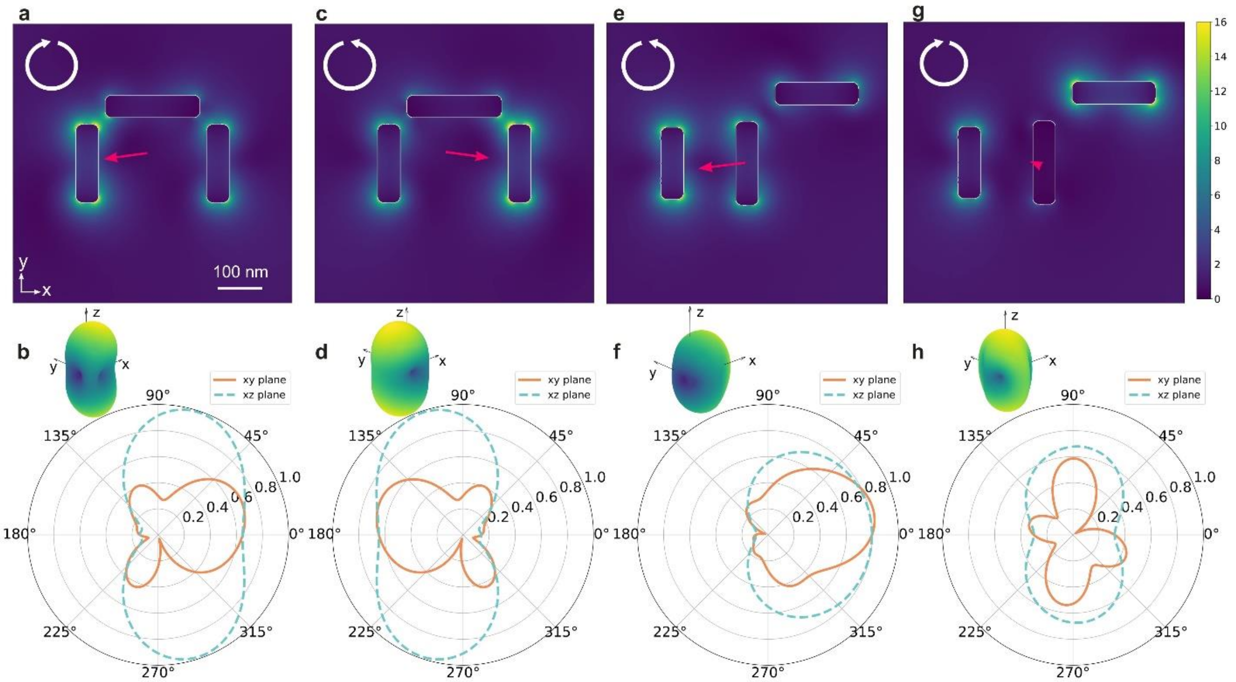

Figure S4. Working principle of plasmonic nanomotors. (a-d), The plasmonic

nanomotor utilized in the two-motor design microrobots is designed to exhibit a chiral response at a wavelength of 980 nm. Panels (**a**) and (**c**) display the electric near-field enhancement when illuminated with CW and CCW polarizations, respectively. Depending on the helicity of the photons, one of the two vertical bars will be resonantly addressed. Panels (**b**) and (**d**) depict the normalized far-field radiation patterns indicating the direction of photon scattering with CW and CCW polarization lasers in the xy and xz planes. Additionally, 3D far-field radiation patterns are shown in the top-left insets. The radiation patterns exhibit high asymmetry in the xy and xz planes, resulting in a directed total optical force generated from photon recoil (labeled by red arrows in (**a**) and (**c**)). **(e-h),** Nanomotors used in the four-motor design microrobots. Electric near-field enhancement and normalized far-field radiation patterns with CW (**g-h**) and CCW (**e-f**) polarizations are depicted. In this design, the nanomotor exhibits good photon scattering directivity with CCW polarization, as evidenced in (**f**), resulting in a large lateral optical force in -x direction. Conversely, in the CW case (**h**), the scattering pattern is symmetric, leading to nearly zero net lateral optical force. The radiation pattern is normalized to the maximum. Scale bar: 100 nm.

More demonstrations of mobile trapping effects with EO modulators (S6)

We implemented another four demonstrations to showcase the microrobot's capabilities of cargo delivery in Fig. S5. Several EO modulators were applied into the optical path to rapidly change the intensities and polarizations of laser beams, without blocking the beams.

In Fig. S5a, we demonstrated translational motion with a 4-motor microrobot. The microrobot's movement direction can be easily altered via EO modulators while maintaining plasmonic trapping effects. In the final three frames, we show back-and-forth translational motion without releasing the trapped nanodiamond. However, the trajectories remain tortuous due to significant Brownian motion and finite laser spot sizes.

In Fig. S5b, using a single laser, we demonstrated complex maneuverability with a 2-motor microrobot controlled via EO modulators. The microrobot followed a polyline path and returned along the same route (indicated by green arrows). While the microrobot's upward or downward motion depends on the helicity of circularly polarized light, its orientation is influenced by spin momentum transfer and Brownian motion. Despite these factors, the microrobot was able to move along arbitrary trajectories without compromising trapping effects.

In Fig. S5c, we demonstrate a typical nanodiamond pickup experiment within a larger working range. In this case, we use nearly maximum output power of our laser diode. In the first two frames, the microrobot captures a single nanodiamond and moves along a larger circular trajectory. Meanwhile, within the dashed white circles, another nanodiamond appears, fluctuating due to Brownian motion. When the microrobot approaches, it captures the second nanodiamond and continues its motion. This demonstrates the microrobot's ability to extend its functionality beyond single nanodiamond trapping, enhancing its working efficiency.

In Fig. S5d, we demonstrate a bio-related application by using the microrobot to capture a single rod-shaped bacterium (*Pseudomonas*) and transport it to arbitrary positions. Interestingly, the bacteria used in this experiment were not intentionally cultivated but resulted from contamination in our lab's water supply. In this case, the microrobot captures one end of the bacterium, drives it to rotate, and performs translational motion. When the laser is switched off, the bacterium detaches from the microrobot immediately.

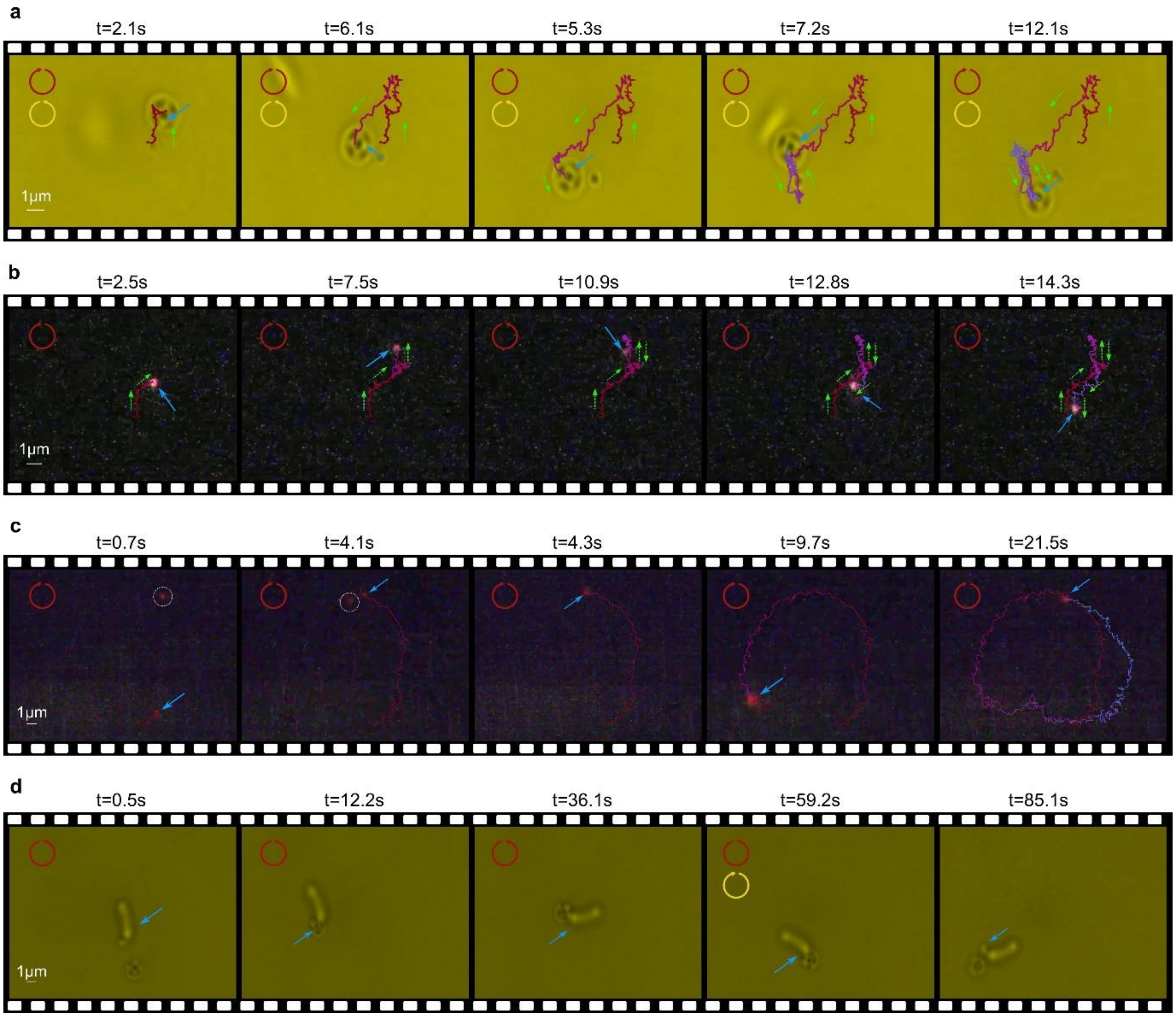

Figure S5. Additional demonstrations of the mobile plasmonic tweezer effect. (a) Bright-field frame sequences (Episode 1 in Supplementary Movie 8) showing translational motion of the 4-motor microrobot driven by two laser beams. By rapidly switching the laser helicities (within a few milliseconds), the microrobot demonstrates back-and-forth motion without releasing the trapped nanodiamond. (b) Fluorescence frame sequences ((Episode 2 in Supplementary Movie 8)) showcasing the complex maneuverability of the 2-motor microrobot within a 980 nm laser spot. The microrobot is

manipulated to follow a polyline path and then return along the same trajectory. The green arrow represents the microrobot's moving direction. (c) Bright-field frame sequences (Episode 3 in Supplementary Movie 8) showing a two-motor microrobot operating within a single laser beam. Initially, the microrobot captures a single nanodiamond and rotates around the laser spot. When the microrobot approaches a free-moving nanodiamond (indicated by the white dashed circle), it successfully captures both nanodiamonds simultaneously. (d) Application in capturing a rod-shaped bacterium (Pseudomonas, Episode 4 in Supplementary Movie 8). In the first frame, the microrobot moves closer to the bacterium, capturing one end of it in the second frame. The microrobot then rotates and performs translational motion while holding the bacterium. In the final frame, when the laser is switched off, the microrobot releases the bacterium, detaching it immediately. Scale bar: 1 μm.

Motion Dynamics (S7)

The angular velocity (translation velocity) is determined by analyzing the bright field video using linear fitting of the total rotation angle (distance moved) over time sequences, as illustrated in Fig. S6. Since the microrobot moves at a uniform velocity in both rotation and translation, the optical thrust force (torque) should balance the drag force. The motion dynamics of the microrobot can be described as planar Couette flow, with a flow Reynolds number of approximately 1.68×10^{-5}. For rotation, the torque caused by the drag force can be evaluated by integrating the shear stress multiplied by the distance to the center over the disk surfaces, as given by the equation $T = \frac{\pi \mu \omega R^4}{2\delta}$. From the FDTD simulations, the optical torque is 0.64 $pN \cdot \mu m$ when applying an optical intensity of 3 $mW/\mu m^2$. By substituting the pure water viscosity μ (typically is $10^{-3} Pa \cdot s$), the radius of the microrobot R (1.75 μm), and the spacing distance between the microrobot and substrate $\delta \approx$ 300 nm, an angular velocity of 9.3 rad/s is expected. The translation velocity, calculated from the drag force equation $F = \frac{\mu A v}{\delta}$, is 23.8 μm/s when the drag force is balanced with optical force of 0.9 pN, which is estimated from simulations with an optical intensity of 3 $mW/\mu m^2$. However, both velocities are found to be 3-5 times larger than the experimental results. This discrepancy can be attributed to the idealized nature of the planar Couette flow model used to describe our experiments. Specifically, the model only considers shear stress from the bottom side of the microrobot for the drag force, while neglecting contributions from the top and side facets. Meanwhile, another significant factor contributing to the drag force is neglected: the pressure force arising from the pressure differential between the front and back facets of the microrobot's body. In future research, a more accurate representation of the motion dynamics can be achieved by employing computational fluid dynamics (CFD) software such as COMSOL.

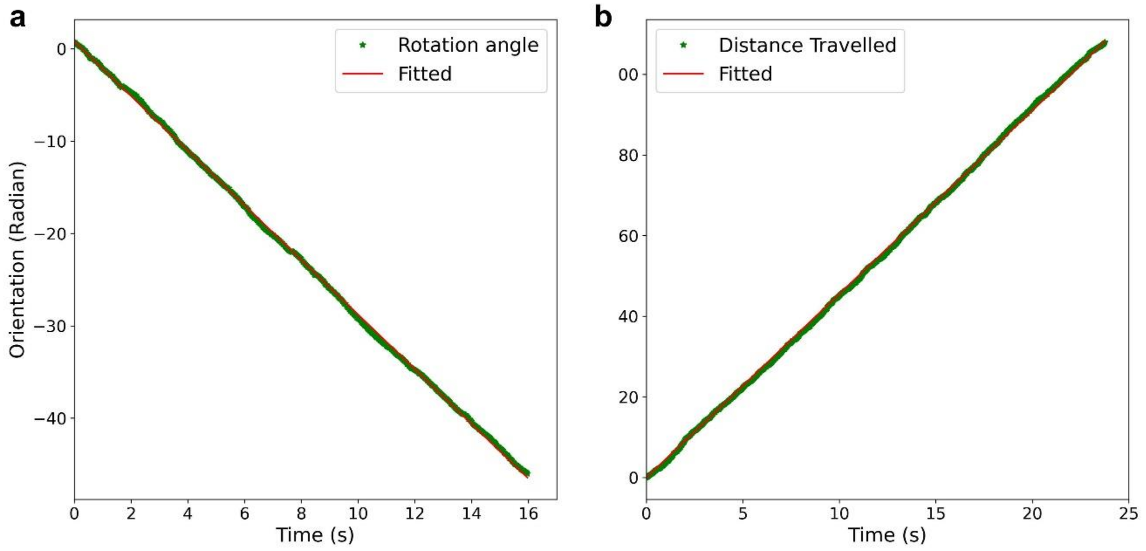

Figure S6: Moton dynamics analysis of microrobots. a. Microrobot rotation angle along with time frames are extracted from image sequences (Supplementary Movie 2). The fitted equation is $\omega(t) = -2.96t + 0.81$. (unit: rad/s) **b**. Microrobot moving distances along with time frames are extracted from image sequences (Supplementary Movie 4). The fitted equation is $v(t) = 4.55t$. (unit: μm/s). Some kinks in the curves show deceleration and acceleration processes of the microrobot when it moves to the margin of the laser spot.

The position accuracy of the nanodiamond trapped by the microrobot was evaluated by fitting the fluorescence trajectories to a circular shape, as shown in Fig. 4b. This approach was used because the bright-field videos make it difficult to accurately determine the coordinates of the trapped nanodiamond. The position accuracy in the radial and azimuthal directions was then calculated based on the mean standard deviation of residuals between the real trajectory and the fitted circle. The residuals as shown in the histograms in Fig.S7b-c were calculated by obtaining the square of the displacement in each movie frame between the r and φ coordinates of the actual position of the fluorescence spot and a point on the circle calculated by assuming a constant angular velocity. The mean standard deviation of the residues was determined by applying a Gaussian fit.

As shown in Fig. S7, the position accuracies in the radial and azimuthal directions were estimated to be 858.77 nm and 14.41 degrees, respectively. Compared to the static tweezer trapping experiments, these values are significantly larger, confirming that the position accuracy is primarily determined by the Brownian motion of the microrobots.

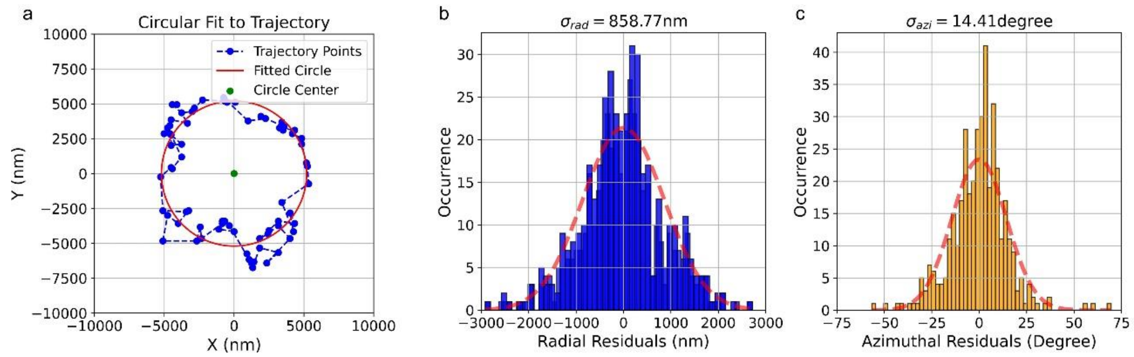

Figure S7. The position accuracy of a nanoparticle trapped by microrobots. (a) Circular fitting of the real trajectories during one complete rotation of the microrobot in Fig. 4b. (b-c) Standard deviations of the residuals between the real trajectories and the fitted circle in the radial and azimuthal directions.

Heat (S8)

The evaluation of plasmonic thermal effects induced by the gold cross antenna is performed using COMSOL Multiphysics to investigate any undesired fluid motion that may affect the trapped nanodiamond. Typically, the thermophoretic force induced by a temperature gradient pushes nanoparticles from higher to lower temperatures. In our simulation, a model similar to our device is constructed, with the antenna partially embedded in silica and the entire device submerged in water. The simulation involved two steps: first, a circularly polarized laser beam with a wavelength of 980 nm and intensity of 3 $mW/\mu m^2$ is used to excite the antenna. The optical absorption of the gold antenna is then treated as a heat source, and the temperature distribution is studied using the thermal diffusion equation[5–7]: $\frac{\rho C \partial T}{\partial t} + \nabla \cdot [\kappa(\boldsymbol{r})\nabla \mathrm{T}(\boldsymbol{r})] = -p(\boldsymbol{r})$, where ρ is the material density, C is heat capacity, κ represents thermal conductivity and $p(\boldsymbol{r})$ is the power density of the heat source. The parameters used for the heat transfer model in the simulation are listed in Table S1.

Figure S8 illustrates the temperature increase distribution ΔK on the XY plane and YZ plane, extracted from a steady-state solution. The blue arrow line represents the temperature gradient, pointing towards higher temperatures. The thermophoretic force, which tends to push nanoparticles from the antenna center (higher temperature) towards the outside (lower temperature), poses a challenge to our trapping effects. Moreover, with the current laser intensity, the temperature increase near the antenna region has reached approximately 50 K, which does not affect stable trapping, but adds more fluctuations. Therefore, it should be noted that higher excitation laser power is not always beneficial for plasmonic trapping, as the total heating power is proportional to the incident laser intensity: $Q = \int d\boldsymbol{r} p(\boldsymbol{r}) = \sigma_{abs} I_0$.

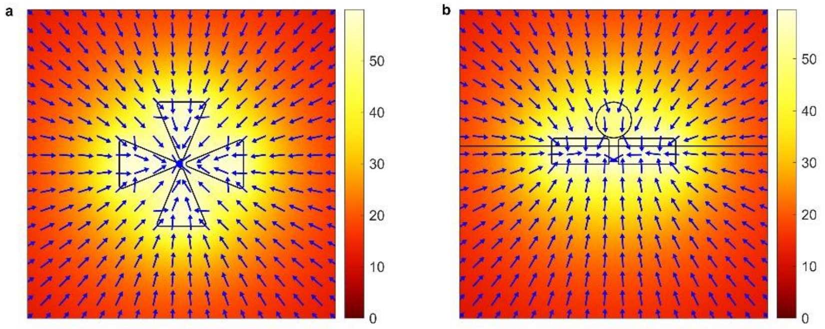

Figure S8: Steady-state heat distribution (ΔT in Kelvin) of the gold cross antenna is simulated using the laser intensity employed in our experiments (3 mW/um^2). The blue arrows consistently indicate the direction from lower to higher temperatures.

	$\rho(kg/m^3)$	$C(J \cdot kg^{-1} \cdot K^{-1})$	$\kappa(W \cdot m^{-1} \cdot K^{-1})$
Gold	19300	126	314
Water	1000	4184	0.58
Silica	2650	680	1.38
Nanodiamond	3500	505	8.5

Table S1: Material properties used in heat transfer simulations.

A rough estimation of the thermophoretic force on nanodiamonds during static trapping experiments was also conducted. Generally, the thermophoretic force is expressed as $F_{thermal} = -k_B T(r) S_T \nabla T(r)$, where k_B is the Boltzmann constant, $T(r)$ is the temperature and S_T is the Soret coefficient of nanoparticles. Although there are few experimental reports on the exact value of the Soret coefficient for nanodiamonds, it can be expressed as follows[8,9]:

$$S_T = -\frac{2\pi R}{k_B T^2}\left[\frac{2\Lambda_l}{2\Lambda_l + \Lambda_p}\right](\varepsilon + T\frac{\partial \varepsilon}{\partial T})\xi^2$$

Here, R is the nanoparticle radius, k_B is Bolzman constant, and Λ_l and Λ_p are the thermal conductivities of the water and nanodiamond, respectively. The thermal conductivity of nanodiamond is set to be 8.5 W/(m·K) from the reference[10]. The static permittivity of water and its temperature derivative $\partial\varepsilon/\partial T$ are obtained from the reference[11]. The zeta potential of the solution ξ is taken as -72.1mV[12].

Besides, the motion of nanoparticles close to the cross antenna is expected to be significantly influenced by hydrodynamic boundary effects, which can increase the Soret factor by an enhanced factor[13]:

$$\Phi_H = 3(1 + H)\frac{(2 + 6H + 3H^2)\ln\left(\frac{H + 1}{H}\right) - 1.5(3 + 2H)}{2 + 9H + 6H^2 - 6H(1 + H^2)\ln\left(\frac{H + 1}{H}\right)}$$

where $H = h/R$ and h is the nanoparticle-surface distance.

Using these equations, we compared the thermophoretic and optical forces acting on nanodiamonds during trapping, as shown in Fig. S9. For the in-plane force distributions (Fig. S9a–c), the thermophoretic force is smaller but of the same order as the optical force. For the out-of-plane force distributions (Fig. S9d–f), the thermophoretic force is five times smaller than the optical force. As a result, the in-plane optical trapping potential is lifted by roughly 1 $k_B T$, mainly due to thermophoresis, leading to a reduction in in-plane trapping stiffness. Nevertheless, the trapping effect clearly prevails because the optical force dominates.

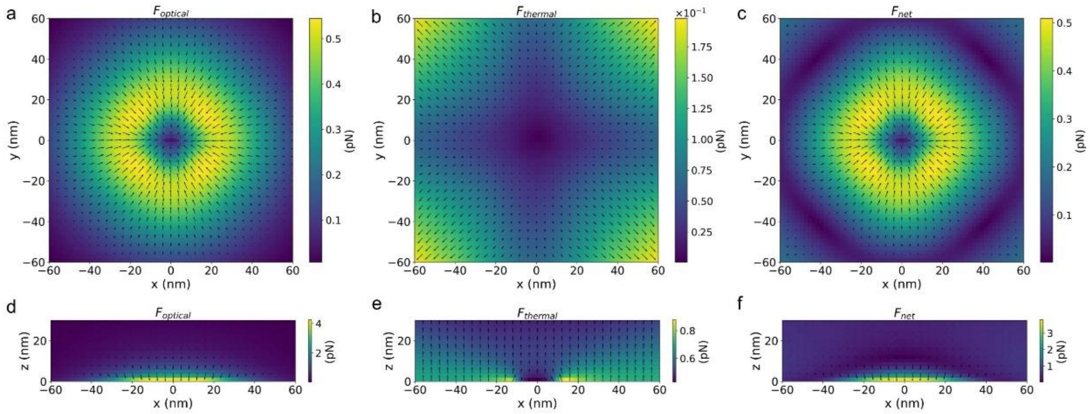

Fig. S9 In-plane and out-of-plane optical, thermophoretic and net force. (a) In-plane optical force for trapping a 70 nm nanodiamond, calculated using the Maxwell Stress Tensor (MST) method, with a laser intensity of $3mW/\mu m^2$. (b) In-plane thermophoretic force distribution calculated based on the local temperature gradient and Soret coefficient of nanodiamonds. (c) In-plane net force, which combines the optical and thermophoretic forces. (d) Out-of-plane optical force distribution. (e) Out-of-plane thermophoretic force distribution. (f) Out-of-plane net force, combining optical and thermophoretic forces. Black arrows in all plots indicate the direction of the forces.

Influence of instrument response on trapping stiffness (S9)

The instrument response is determined by monitoring the fluorescence of a single nanodiamond which adheres to the coverslip. To achieve this, an aqueous nanodiamond suspension is spin-coated onto a clean coverslip, ensuring attachment of the nanodiamond to the coverslip surface after evaporation of the water. The experimental setup depicted in Fig. S2b is then employed to record the fluorescence emitted by the immobilized single nanodiamond. The same methods are used to process the image sequences and extract the center position as were described above to investigate the trapping. Through multiple

measurements, the instrument response in the x and y directions is determined to be 28.8 ± 8.5 nm and 26.3 ± 9.3 nm, respectively, as shown in Fig. S10a. It should be noted that these variations may arise due to shot noises, saturation of color center emission, or slight drift in the measurement system. Additionally, accounting for the temperature increase in the surrounding area caused by plasmonic heating (T=340 K) leads to higher trapping stiffness values in static tweezer experiment, with k_x and k_y reaching up to 2.70 ± 1.28 fN/nm and2.65 ± 1.28 fN/nm, respectively.

The trapping stiffness can also be evaluated by taking the derivative of the optical force distribution (Fig. 2e) with respect to the displacement around the equilibrium point, expressed as $k_i = \frac{\partial F_i}{\partial X_i}$[14]. This theoretical evaluation yields the upper limit of trapping stiffness value of approximately 25 fN/nm, as shown in Fig. S10b. This maximum trapping stiffness only is valid within a small regime (roughly ±20nm), where the optical force is linear. However, in the experiments (Fig. 3c-d), the coordinates position can vary within ±150nm, where the optical force starts to decrease and effective trapping stiffness is much smaller. This discrepancy could also potentially be attributed to the faceted and not entirely symmetric shape of the nanodiamond.

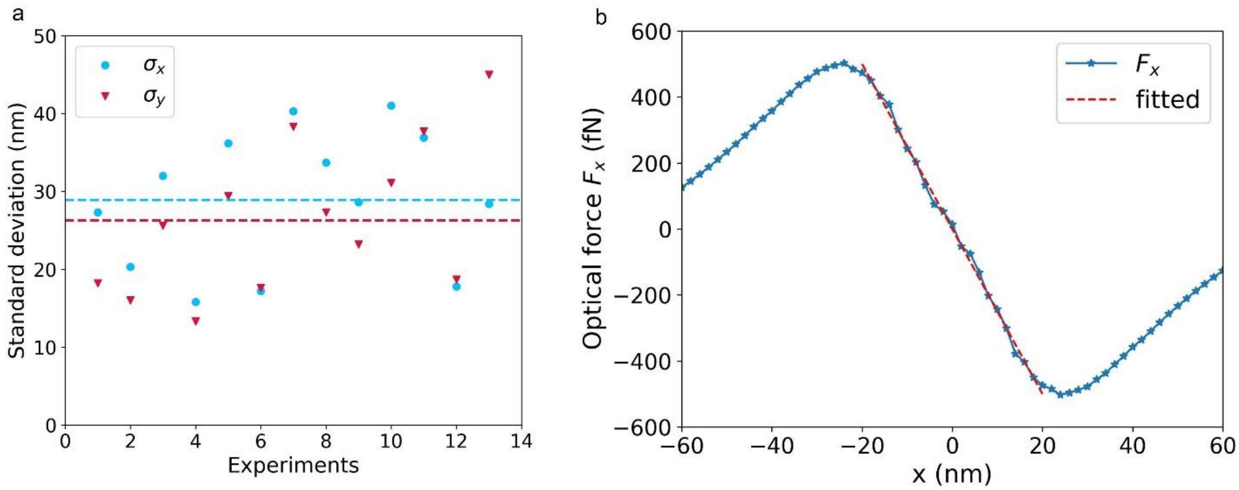

Figure S10: Statistical Analysis of Instrumental Responses in the X(Y) Directions and theoretical evaluations of trapping stiffness. (a)The blue and red dashed lines represent the averaged values obtained from multiple measurements. (b) The optical force distribution in x direction (y=0 nm) from Fig. 2e. Trapping stiffness is obtained by fitting the linear regime of optical force within a small regime as indicated by red dashed line.

References

1. Lindner, S. *et al.* Strongly inhomogeneous distribution of spectral properties of silicon-vacancy color centers in nanodiamonds. *New J. Phys.* **20**, 115002 (2018).

2. Sigaeva, A., Merz, V., Sharmin, R., Schirhagl, R. & Krueger, A. Intracellular behavior of nanodiamonds functionalized with a zwitterionic shielding moiety. *J. Mater. Chem. C* **11**, 6642–6650 (2023).

3. Merz, V. *et al.* Zwitterion-Functionalized Detonation Nanodiamond with Superior Protein Repulsion and Colloidal Stability in Physiological Media. *Small* **15**, 1901551 (2019).

4. Wu, X. *et al.* Light-driven microdrones. *Nat. Nanotechnol.* **17**, 477–484 (2022).

5. Baffou, G. & Quidant, R. Thermo-plasmonics: using metallic nanostructures as nano-sources of heat. *Laser Photonics Rev.* **7**, 171–187 (2013).

6. Baffou, G., Quidant, R. & García de Abajo, F. J. Nanoscale Control of Optical Heating in Complex Plasmonic Systems. *ACS Nano* **4**, 709–716 (2010).

7. Richardson, H. H., Carlson, M. T., Tandler, P. J., Hernandez, P. & Govorov, A. O. Experimental and Theoretical Studies of Light-to-Heat Conversion and Collective Heating Effects in Metal Nanoparticle Solutions. *Nano Lett.* **9**, 1139–1146 (2009).

8. Putnam, S. A., Cahill, D. G. & Wong, G. C. L. Temperature Dependence of Thermodiffusion in Aqueous Suspensions of Charged Nanoparticles. *Langmuir* **23**, 9221–9228 (2007).

9. Anderson, J. L. Colloid Transport by Interfacial Forces. *Annu. Rev. Fluid Mech.* **21**, 61–99 (1989).

10. Kidalov, S. V., Shakhov, F. M. & Vul, A. Ya. Thermal conductivity of sintered nanodiamonds and microdiamonds. *Diam. Relat. Mater.* **17**, 844–847 (2008).

11. Archer, D. G. & Wang, P. The Dielectric Constant of Water and Debye-Hückel Limiting Law Slopes. *J. Phys. Chem. Ref. Data* **19**, 371–411 (1990).

12. Hong, C., Yang, S., Kravchenko, I. I. & Ndukaife, J. C. Electrothermoplasmonic Trapping and Dynamic Manipulation of Single Colloidal Nanodiamond. *Nano Lett.* **21**, 4921–4927 (2021).

13. Würger, A. Hydrodynamic Boundary Effects on Thermophoresis of Confined Colloids. *Phys. Rev. Lett.* **116**, 138302 (2016).

14. Rohrbach, A. Stiffness of Optical Traps: Quantitative Agreement between Experiment and Electromagnetic Theory. *Phys. Rev. Lett.* **95**, 168102 (2005).
