# Supplementary material for: Light-driven plasmonic microrobot for nanoparticle manipulation
Source: Nat Commun. 2025 Mar 15;16:2570. doi: 10.1038/s41467-025-57871-x (PMC11910605; doi:10.1038/s41467-025-57871-x)
Supplement: Supplementary file 2 — Description of Additional Supplementary Files [file 41467_2025_57871_MOESM2_ESM.pdf]

Description of Additional Supplementary Files

Supplementary Movie 1: Static tweezer experiment.

Supplementary Movie 2: Rotation motion of the microrobot with a trapped nanodiamond (Bright field)

Supplementary Movie 3: Rotation motion of the microrobot with a trapped nanodiamond (Fluorescence)

Supplementary Movie 4: Translation motion of the microrobot with a trapped nanodiamond (Bright field)

Supplementary Movie 5: Translation motion of the microrobot with a trapped nanodiamond (Fluorescence)

Supplementary Movie 6: Entire trapping dynamics with microrobot doing rotation motion.

Supplementary Movie 7: Manipulation of the microrobot within two wavelengths while maintaining the trapping effects. (Episode 1: Bright-filed and Episode 2: Fluorescence)

Supplementary Movie 8: Another 4 demonstrations for showing the capabilities of microrobots.
(Episode 1: A 4-motor microrobot driven by two laser beams with EO modulators (Bright Field). Episode 2: Complex maneuverability of a 2-motor microrobot driven with EO modulators (Fluorescence). Episode 3: A 2-motor microrobot operating within an extended dynamic range (Fluorescence). Episode 4: A microrobot capturing, transporting, and releasing a single bacterium (Bright Field).)
